# Single cell transcriptome revealed SARS-CoV-2 entry genes enriched in colon tissues and associated with coronavirus infection and cytokine production

**DOI:** 10.1038/s41392-020-00237-0

**Published:** 2020-07-08

**Authors:** Haoyan Chen, Tian-Hui Zou, Baoqin Xuan, Yuqing Yan, Tingting Yan, Chaoqin Shen, Gang Zhao, Ying-Xuan Chen, Xiao Xiao, Jie Hong, Jing-Yuan Fang

**Affiliations:** 1 State Key Laboratory for Oncogenes and Related Genes, Division of Gastroenterology and Hepatology, Shanghai Institute of Digestive Disease, Renji Hospital, School of Medicine, Shanghai Jiao Tong University, 145 Middle Shandong Road, Shanghai, 200001 China; 2 Department of Gastrointestinal Surgery, Renji Hospital, School of Medicine, Shanghai Jiao Tong University, Shanghai, 200127 China

**Keywords:** Cell biology, Gastroenterology

**Dear Editor,**

The novel COVID-19 coronavirus (SARS-CoV-2) endangers thousands of lives. Recently, nearly 10% COVID-19 patients with symptoms of the digestive tract were subsequently diagnosed in newly discovered SARS-CoV-2 infected cases,^1^ which indicates that SARS-CoV-2 may infect people via digestive systems. The expression profile of angiotensin-converting enzyme homolog 2 (ACE2) has been analyzed in gastrointestinal tract using external datasets. Unfortunately, most data of these datasets are not from Asian adults and the expression of other SARS-CoV-2 entry genes have not been detected in human gastrointestinal tract. Therefore, to provide more implications of viral transmission in clinical diagnosis and treatment, it is urgent to explore the expression of SARS-CoV-2 entry genes in the gastrointestinal tract of Asian people. Here, we used single-cell RNA sequencing profiled ~27,800 cells of intestinal tissues from six patients with basic intestinal disease of Renji hospital.

## A single-cell atlas and cell typing of six patients with basic intestinal disease of Renji hospital

To characterize the single-cell profile of colon mucosae, a total of six biopsies, including two adjacent normal tissue, two patients with colorectal adenoma (CRA) biopsies, and two patients with colorectal cancer (CRC) biopsies, were taken from six patients, who spanned the cascade from normal colon, adenoma to colorectal cancer (Supplementary Table S1). For each biopsy, we isolated single cells without prior selection for cell types and used the 10x Chromium platform to generate RNA-seq data. After removing low quality cells, a total of 27,809 cells that passed quality control were retained for subsequent analysis (Supplementary Fig. S1a). The number of cells from each biopsy was provided in Table S1. The global expression profiles and shifts in cell-intrinsic programs and cell proportions were illustrated in these six patients (Supplementary Fig. S1b, c).

The distributions of different cell types, which includes epithelial cells, fibroblasts, and other immune cells were presented as two-dimensional (2D) UMAP plot in normal colon tissues, colorectal adenoma and colorectal cancer tissues (Supplementary Fig. S2a). Following gene expression normalization for read depth and mitochondrial read count, we applied principle component analysis on genes variably expressed across all 27,809 cells (*n* = 23,877 genes). Subsequently, we classified cells into groups of cell types using graph-based clustering on the informative principle components (*n* = 50). This identified cell clusters that, through marker genes, could be readily assigned to known cell lineages: in addition to cancer cells, we identified immune cells (CD4+ T cells, B cells, CD8+ T cells, dendritic cells, macrophage, and plasma cells), fibroblasts, and epithelial cells (Supplementary Fig. S2b–k). We further classified all the cells in normal control, CRA, CRC samples by eight major colon cell types using cell-type specific markers and showed that epithelia were the most abundant cell type (40.2 ± 16.1%) in all the samples, followed by other cell types such as T cells (24.1 ± 11.1%) and plasma cells (Supplementary Fig. S3a). To identify which cells type was the major candidate target for SARS-CoV-2 infection, we examined the six potential SARS-CoV-2 entry genes in all cell populations. The cells with ACE2 expression >0 were considered as ACE2-positive cell. We observed that percentage of ACE2-positive cell is gradually increased from normal control, CRA to CRC samples (Supplementary Fig. S3b), and this receptor is mostly enriched in epithelial cells among different cell types of three kinds of colorectal samples (Supplementary Fig. S3c). Next, the expression of CD147, TMPRSS2, CTSB (CATB), CTSL (CATL), and Furin was detected in epithelial cells of normal control, CRA, and CRC samples. The percentage of these five SARS-CoV-2 entry genes was similar as ACE2, which is gradually increased in epithelial cells from normal control, CRA to CRC samples (Supplementary Fig. S3d). Our data may partially explain the report of Liang et al. group. They found cancer patients including CRC, might have a higher risk of SARS-CoV-2 than individuals without cancer.^2^

## The pathways that co-expressed with potential SARS-CoV-2 entry genes

**Fig. 1:**
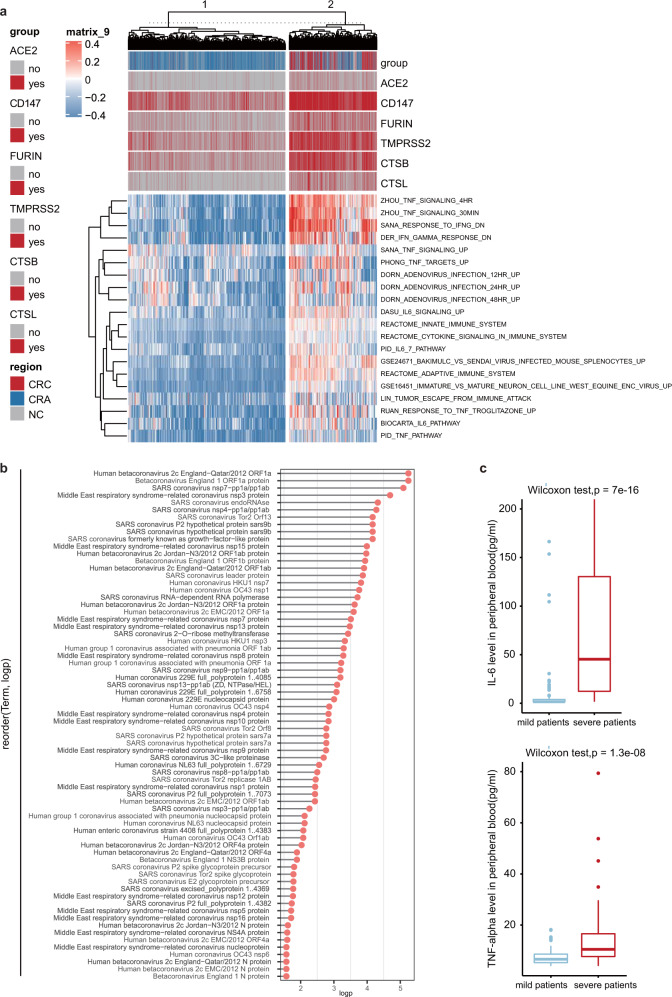
Six potential COVID-19 entry genes co-expressed pathways. **a** Heatmap showing the expression of the MSigDB defined pathway epithelial cells from human normal colon tissue, adenoma and colorectal cancer. **b** Cleveland dot plot showing ACE2 co-expressed virus–host interaction pathways. **c** Box plot showing IL-6 and TNF-alpha status in peripheral blood of 152 patients with COVID-19 pneumonia patients

To identify global pathways that may co-express with six potential SARS-CoV-2 entry genes, we performed single-sample Gene Set Enrichment Analysis, or ssGSEA, to calculate the enrichment scores of predefined pathways on epithelial cells. Strikingly, pathways associated with virus infection, inflammation and cytokine signaling were upregulated in six potential SARS-CoV-2 entry genes enriched cells (Fig. 1a). We also observed that those pathways were prominent on the cells with higher expression of SARS-CoV-2 entry genes. ssGSEA analysis on external dataset GSE97693 revealed consistent results (Supplementary Fig. S4a). Given that ACE2 plays major roles in mediating viral entry, we performed Spearman correlation analysis between ACE2 and other genes. In total, 271 genes (*p* < 0.01) were positively with ACE2 expression. By using Gene Ontology (GO) analysis on Enrichr software, we observed that genes positively correlated with ACE2 expression were enriched in virus infection, transmission, and virus–host interaction pathways (Fig. 1b, Supplementary Fig. S4b, Supplementary Table S2).

## IL-6 and TNF-alpha in peripheral blood of COVID-19 pneumonia patients

Given that IL-6 and TNF pathways were upregulated in six potential SARS-CoV-2 entry genes enriched cells (Fig. 1a), we compared the IL-6 and TNF-alpha levels in peripheral blood from mild (*n* = 102) and severe (*n* = 50) COVID-19 pneumonia patients (Supplementary Table S3). We observed that both IL-6 (median: 45.28 versus 1.50, *p*-value = 7E−16) and TNF-alpha (median: 15.44 versus 7.29, *p*-value = 1.3E–8) were significantly higher in severe COVID-19 pneumonia patients than mild samples (Fig. 1c).

A distinct coronavirus SARS-CoV was identified as the etiological agent of severe acute respiratory syndrome (SARS). This virus may destroy the upper respiratory tract and damage the patient’s intestines by causing diarrhea. In December 2019, SARS-CoV-2 from the same family as SARS virus has been identified, and this virus can also cause fever, lower respiratory tract infection and gastrointestinal symptoms.^1^ This virus may directly bind the angiotensin-converting enzyme II (ACE2), which was known as cell receptor for SARS-CoV.^3^ Interestingly, SARS-CoV-2 just specifically uses ACE2 to infect mammal cells, but not other coronavirus receptor.^3^ Recently, a novel cellular receptor of SARS-CoV-2, CD147 has been identified,^4^ and this study revealed that SARS-CoV-2 enter cells may not only just depends on ACE2, but also CD147 receptor. In addition, block the protease activity of TMPRSS2 and the endosomal cysteine proteases cathepin B and L (CatB/L) successfully blocked SARS-CoV-2 entering colon Caco-2 cells.^5^ These studies indicate that SARS-CoV-2 not just depends on ACE2 to infect and transmit in the host body, but also require other SARS-CoV-2 entry genes, such as CD147, TMPRSS2, CatB/L, and Furin.

It is reported that ACE2 and TMPRSS2 are highly expressed in the lung and other digestive tract organs in public external databases. However, Asian adult data is rare in these datasets. Here, we first proved that the six SARS-CoV-2 entry genes, including ACE2 and TMPRSS2, are expressed in the colon epithelial cells of Chinese adults. The expression of these SARS-CoV-2 entry genes is gradually increased from normal colon epithelium, adenoma to colorectal cancer patients’ tissues. Furthermore, we found that those genes, which are closely associated with coronavirus infection, are more likely enriched in SARS-CoV-2 entry genes positive colon cells. Based on the expression profiling and biological pathway analysis of these SARS-CoV-2 entry genes, we speculates that those patients with higher expression of SARS-CoV-2 entry genes, may be more likely to be infected and injured by COVID-19, than healthy people. However, we need more experiments to verify our hypothesis in future exploration.

In a word, the expression of additional five SARS-CoV-2 entry genes, virus infection, and inflammatory pathways are significantly enriched in colon epithelia cells with ACE2-positive expression. Understanding of the tissue tropism of SARS-CoV-2 on intestinal cells may also help to elucidate the pathogenetic mechanism of this virus and possibly help in the development of novel antiviral therapy.

## Supplementary information

Supplementary Materials

Supplementary Figures
