# Supplementary material for: Single cell transcriptome revealed SARS-CoV-2 entry genes enriched in colon tissues and associated with coronavirus infection and cytokine production
Source: Signal Transduct Target Ther. 2020 Jul 8;5:121. doi: 10.1038/s41392-020-00237-0 (PMC7340775; doi:10.1038/s41392-020-00237-0)
Supplement: Supplementary file 1 — Supplementary Materials [file 41392_2020_237_MOESM1_ESM.docx]

Supplementary Materials for

Single cell transcriptome revealed SARS-CoV-2 entry genes enriched in colon tissues and associated with coronavirus infection and cytokine production

Haoyan Chen, Tian-Hui Zou, Baoqin Xuan, Yuqing Yan, Tingting Yan, Chaoqin Shen, Gang Zhao, Ying-Xuan Chen, Xiao Xiaoi, Jie Hong, Jing-Yuan Fang

Correspondence to: jiehong97@sjtu.edu.cn or jingyuanfang@sjtu.edu.cn

**This PDF file includes:**

Materials and Methods

Supplementary Text

Figures. S1 to S4

Tables S1 to S3

Materials and Methods

**Collection of COVID-19 pneumonia patients**

Data of 150 patients with confirmed COVID-19 pneumonia hospitalized at Wuhan Leishenshan Hospital from Feb 24，2020 to April 4th，2020 were retrospectively collected. Patients were diagnosed with COVID-19 pneumonia according to World Health Organization interim guidance. The cytokines including IL-6 and TNF-alpha

were detected by multiple microsphere flow immunofluorescence according to the manufacturer’s instructions. Clinical Trial Center of Zhongnan Hospital of Wuhan University approved the study.

**Ethics approval**

The study has been approved by Ethics Committees of Zhongnan Hospital of Wuhan University and Shanghai Renji Hospital.

**Single cell RNA-sequencing**

Single cell libraries were generated using the Chromium Single Cell 3′ library and gel bead kit v3 from 10x Genomics. Libraries were sequenced on the NovaSeq 6000 System (Illumina) at Genergy Bio-technology (Shanghai, China) Co.,Ltd with 150 bp paired-end sequencing of reads. Gene counts were obtained by aligning reads to the hg38 genome (GRCh38) using CellRanger toolkit (version 3.0.2, 10X Genomics). The unique molecular identifier (UMI) count matrix was converted to anndata objects (version 0.6.22) using the Scanpy package v1.4.4 [12]. We utilized the following procedure to control for data quality: cells with fewer than 200 genes, and genes that are detected in less than 3 cells. Following the quality control procedure, the dataset consisted of 27,809 cells expressing 23,877 genes. The filtered gene expression matrix was normalized using Scanpy’s sc.pp.normalize_total function. A single-cell neighborhood graph was computed on the 50 first principal components that sufficiently explain the variation in the data using 15 nearest neighbors. Uniform Manifold Approximation and Projection (UMAP) was run for visualization. Cell types were annotated based on the expression of known marker genes. Single-sample gene set enrichment analysis (ssGSEA) was used to assess gene set activation scores in gene expression profiling data. The ssGSEA score was calculated by Gene Set Variation Analysis (GSVA) R package.

External data sources: GSE97693 were used for bioinformatics analysis. The transcripts per million mapped reads (TPM) were retrieved from GEO database(<https://www.ncbi.nlm.nih.gov/geo/)>.


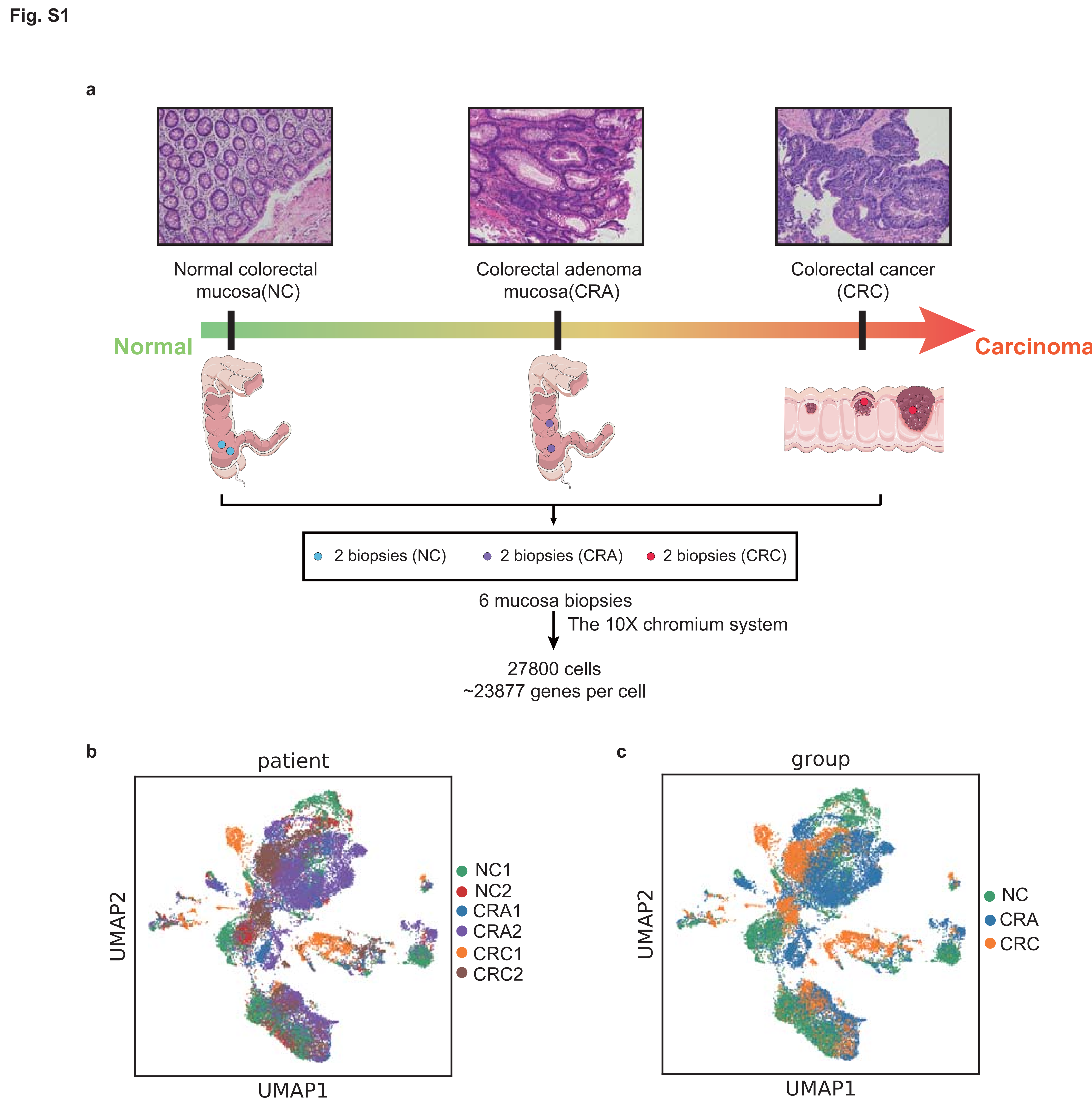


**Fig. S1.** Single-cell RNA-seq of human normal colon tissue, adenoma and colorectal cancer. (a) Schematic workflow of the experimental strategy. (b) UMAP plot of single-cell transcriptomes based on six patients (c) UMAP plot of single-cell transcriptomes based on three group


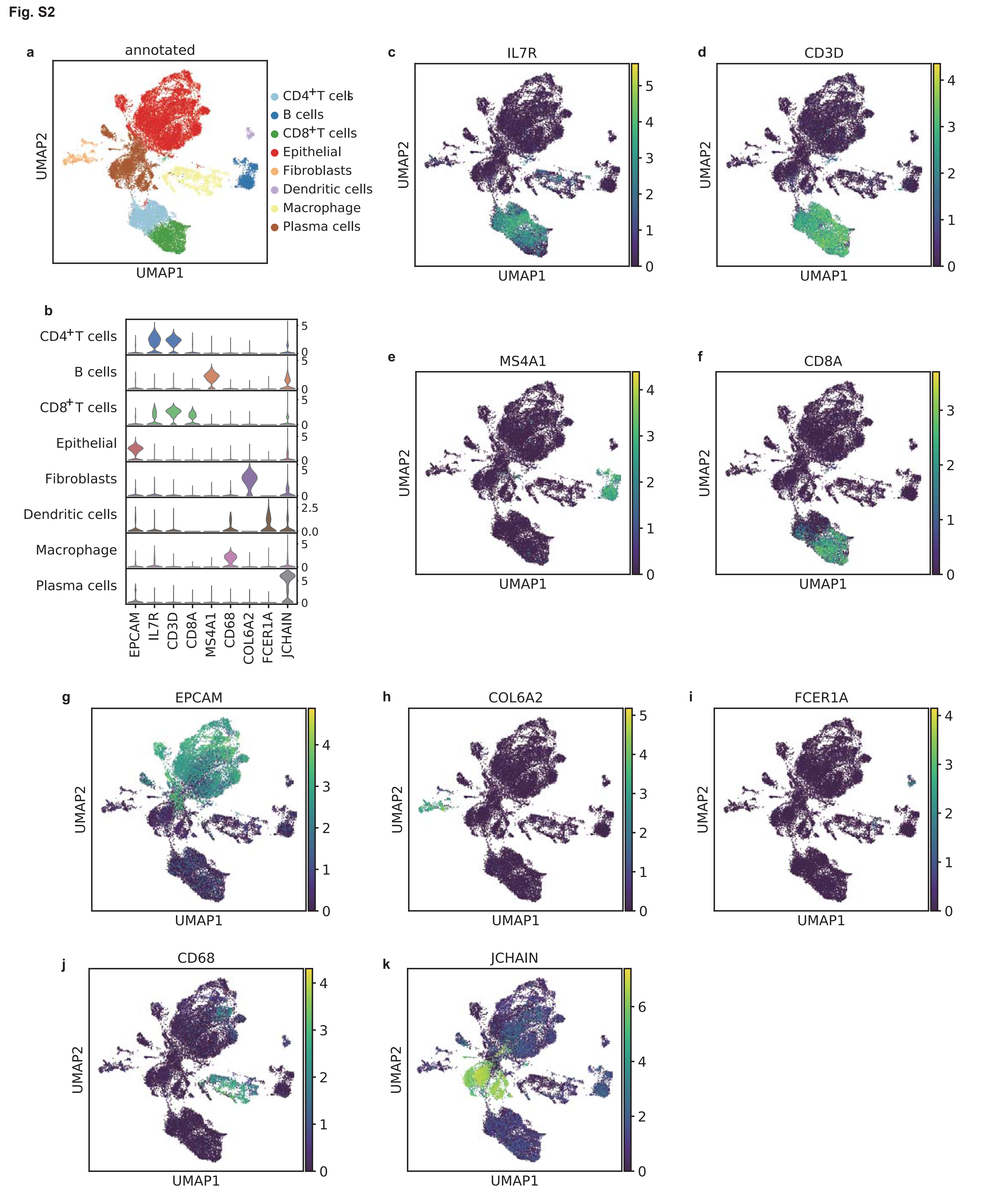


**Fig.S2.** (a) Heatmap showing the expression of the MSigDB defined pathway epithelial cells from GSE97693. (b) Cleveland dot plot showing ACE2 co-expressed GO pathways.


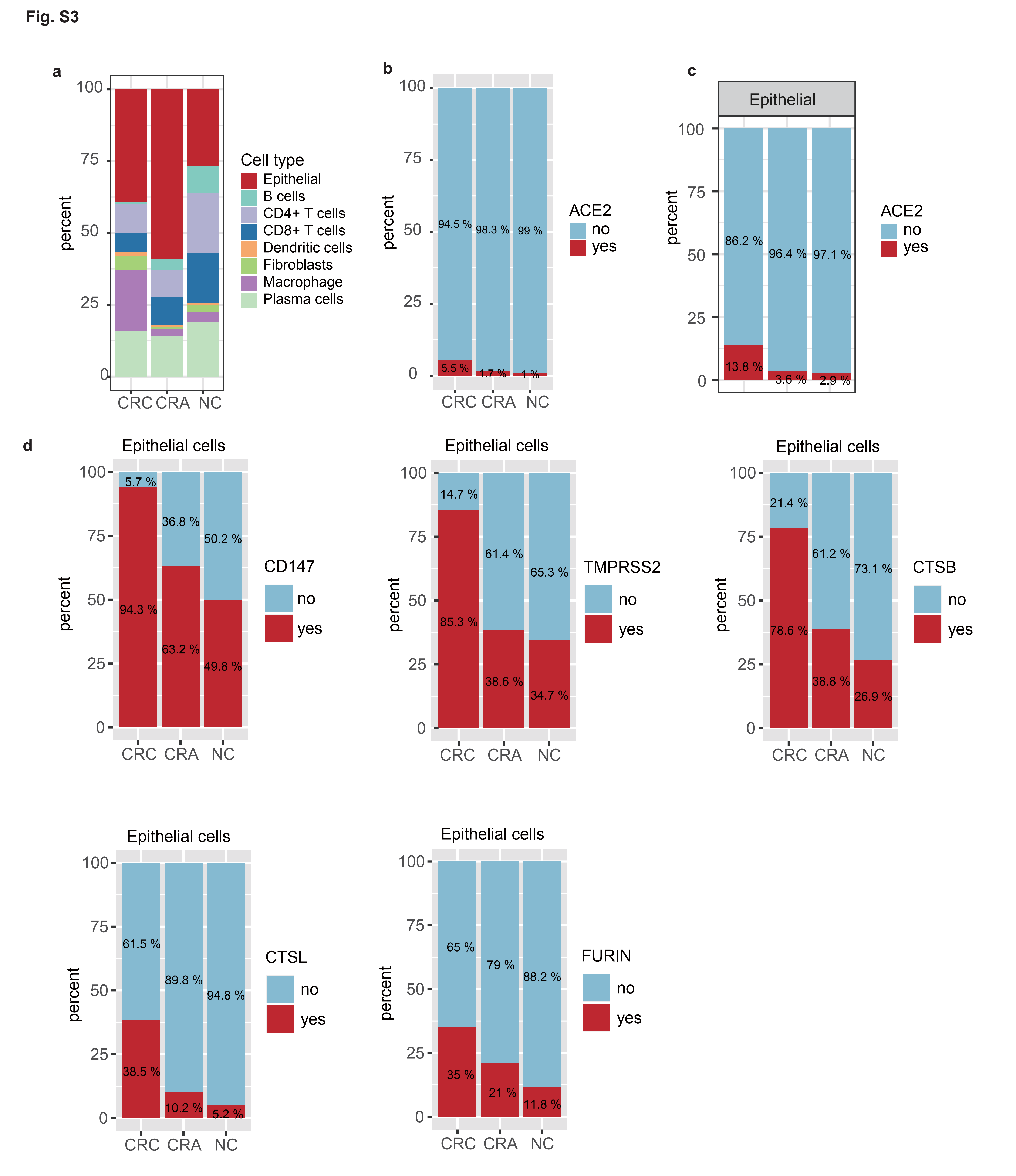


**Fig.S3.** Landscape of cell types in human normal colon tissue, adenoma and colorectal cancer (a) Bar plot showing the cell fraction of cell types that aligned to different group of patients. (b) Bar plot showing the cell fraction of ACE2 positive cell that aligned to different group of patients. (c) Bar plot showing the cell fraction of ACE2 positive cell that aligned to different group of patients in epithelial cell. (d) Bar plot showing the cell fraction of five potential COVID-19 entry genes positive cell in epithelial cells that aligned to different group of patients.


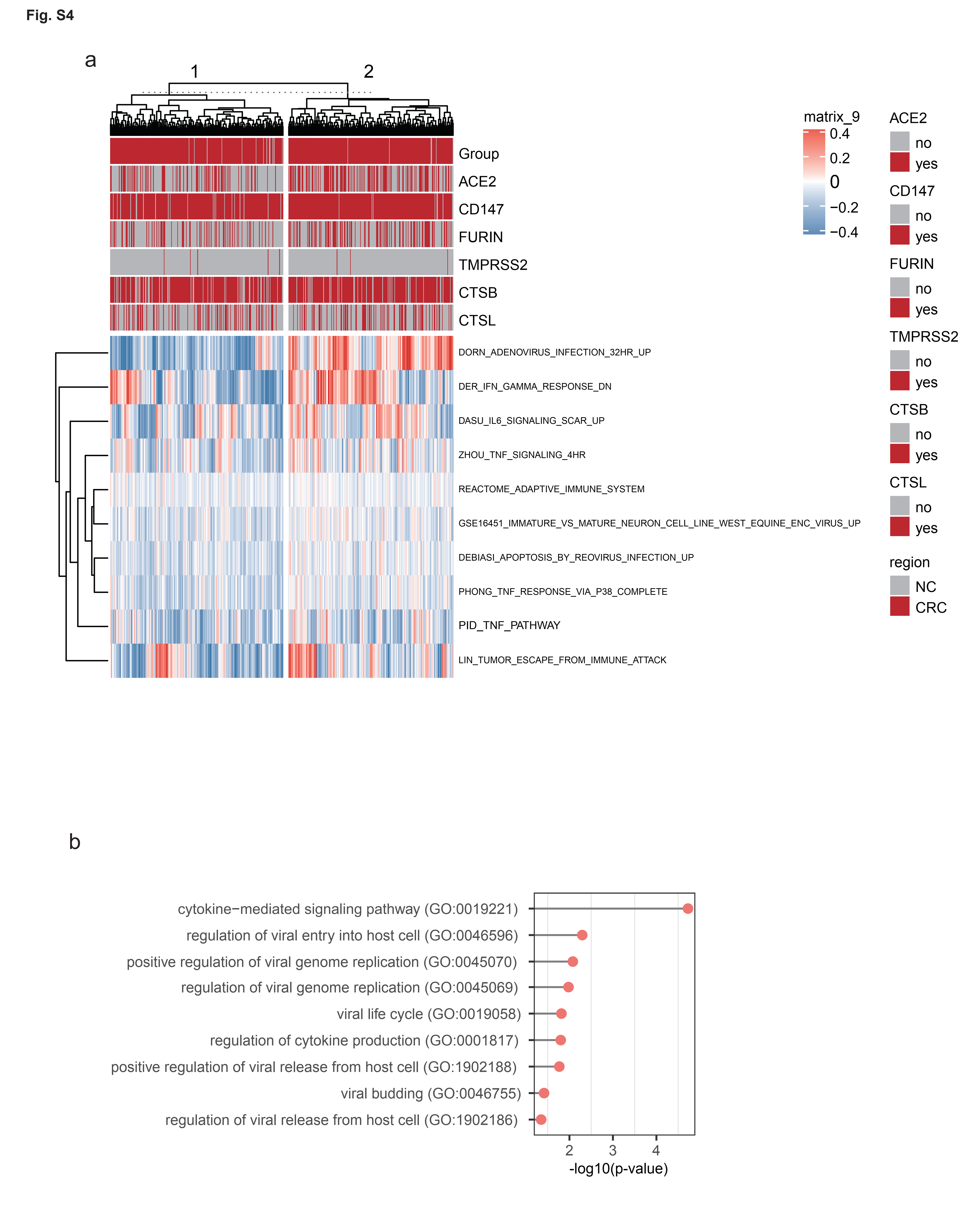
**Fig.S4**. Six potential COVID-19 entry genes co-expressed pathways. (a) Heatmap showing the expression of the MSigDB defined pathway epithelial cells from human normal colon tissue and colorectal cancer on external dataset GSE97693. (b) Cleveland dot plot showing ACE2 co-expressed virus-host interaction pathways.

**Table S1 Clinical characteristics of single cell RNA-sequencing patients**

| Sample ID | Gender | Age | Anatomic Location of Lesion | Pathological diagnosis | Detected Cells |
| --- | --- | --- | --- | --- | --- |
| NC1 | Male | 55 | Transverse Colon | Colon adenoma | 4300 |
| CRA1 | Female | 81 | Sigmoid Colon | Colon adenoma & Colorectal cancer | 7680 |
| CRC1 | Female | 81 | Sigmoid Colon | Colon Adenocarcinoma | 3414 |
| CRA2 | Male | 74 | Ascending Colon | Colon adenoma & Colon Adenocarcinoma | 3601 |
| NC2 | Female | 33 | Descending Colon | Colon adenoma | 6818 |
| CRC2 | Male | 75 | Rectum | Colon Adenocarcinoma | 1996 |

**Table S2 The gene list positively correlates with ACE2 expression**

| Gene symbol | p value | rho |
| --- | --- | --- |
| MT-ND4L | 5.02E-52 | 0.5617462 |
| IGKC | 9.50E-50 | 0.551086549 |
| MT-ND5 | 8.51E-43 | 0.515951226 |
| MALAT1 | 7.81E-32 | 0.450579961 |
| HLA-A | 9.05E-28 | 0.422236415 |
| PHGR1 | 3.35E-27 | 0.418051285 |
| NEAT1 | 8.01E-27 | 0.415225521 |
| SELENOP | 3.34E-26 | 0.410547283 |
| TSPAN1 | 2.56E-25 | 0.40373217 |
| SMIM22 | 4.41E-25 | 0.40188796 |
| CDHR5 | 4.26E-24 | 0.394067127 |
| CA4 | 7.37E-23 | 0.383930614 |
| CTSD | 2.75E-22 | 0.379134303 |
| FXYD3 | 3.30E-22 | 0.378458684 |
| CYP3A5 | 4.24E-22 | 0.37753222 |
| PIGR | 2.48E-21 | 0.370926539 |
| CDC42EP5 | 4.34E-21 | 0.368796005 |
| FCGBP | 9.20E-21 | 0.365912443 |
| CLCA4 | 2.19E-19 | 0.35341915 |
| MT-CO1 | 2.84E-19 | 0.352376402 |
| SLC44A4 | 6.13E-19 | 0.349250539 |
| HLA-E | 7.61E-19 | 0.348369416 |
| UBC | 6.65E-18 | 0.339330817 |
| C19orf33 | 9.30E-18 | 0.337906182 |
| SERINC2 | 2.68E-17 | 0.333356857 |
| HLA-C | 2.93E-17 | 0.332974236 |
| ACADVL | 3.78E-17 | 0.331866418 |
| MT-ND3 | 3.98E-17 | 0.331646566 |
| GUCA2B | 4.00E-17 | 0.33161835 |
| IGLC2 | 5.91E-17 | 0.329912923 |
| MT-CO3 | 8.03E-17 | 0.328568728 |
| SH3BGRL3 | 9.67E-17 | 0.327746619 |
| MYL12B | 1.62E-16 | 0.325450734 |
| DST | 2.04E-16 | 0.324423091 |
| MUC12 | 2.30E-16 | 0.323892638 |
| VSIG2 | 2.50E-16 | 0.323513297 |
| KRT19 | 4.27E-16 | 0.321100211 |
| DUOX2 | 5.04E-16 | 0.320349056 |
| SDCBP2 | 7.11E-16 | 0.318782816 |
| ZG16 | 7.39E-16 | 0.31860645 |
| EDF1 | 9.88E-16 | 0.317275224 |
| ITM2C | 2.41E-15 | 0.313133357 |
| UGP2 | 5.19E-15 | 0.30954041 |
| CDHR2 | 1.82E-14 | 0.30353103 |
| GUCA2A | 2.18E-14 | 0.302663737 |
| MYL6 | 2.44E-14 | 0.302099067 |
| LGALS2 | 6.66E-14 | 0.297167208 |
| EFHD2 | 1.19E-13 | 0.294281593 |
| IGHA1 | 1.84E-13 | 0.292072726 |
| CST3 | 2.89E-13 | 0.289780061 |
| FAM3D | 3.26E-13 | 0.289151122 |
| PRSS3 | 3.37E-13 | 0.288982918 |
| MT1E | 3.58E-13 | 0.288674199 |
| ABCB1 | 1.01E-12 | 0.283305984 |
| RRBP1 | 1.95E-12 | 0.279819271 |
| B2M | 3.26E-12 | 0.277075658 |
| TMIGD1 | 3.71E-12 | 0.276382289 |
| BLOC1S1 | 4.84E-12 | 0.274937732 |
| GSN | 7.64E-12 | 0.272453402 |
| TFF1 | 8.48E-12 | 0.271880276 |
| AHNAK | 1.08E-11 | 0.270527076 |
| CYSTM1 | 1.56E-11 | 0.268494728 |
| SLC6A19 | 2.15E-11 | 0.266714124 |
| LMO7 | 2.99E-11 | 0.264852217 |
| DSC2 | 4.17E-11 | 0.262970497 |
| CD177 | 4.71E-11 | 0.26226508 |
| TCF25 | 4.90E-11 | 0.262048023 |
| GCNT3 | 7.69E-11 | 0.259450059 |
| DUOXA2 | 8.83E-11 | 0.258645981 |
| JUND | 1.06E-10 | 0.257611224 |
| MYL12A | 1.45E-10 | 0.255768843 |
| IFI27 | 3.40E-10 | 0.250680711 |
| KRT20 | 3.89E-10 | 0.249873015 |
| MXD1 | 4.55E-10 | 0.24892585 |
| JUN | 5.38E-10 | 0.247913228 |
| RHOC | 6.68E-10 | 0.246591045 |
| IGHA2 | 6.89E-10 | 0.246404477 |
| MALL | 1.26E-09 | 0.242659042 |
| C11orf86 | 1.47E-09 | 0.241709881 |
| CEACAM7 | 1.69E-09 | 0.240856565 |
| MT-ND1 | 2.32E-09 | 0.238839881 |
| CA1 | 2.46E-09 | 0.238464909 |
| TMEM59 | 3.87E-09 | 0.235589316 |
| EZR | 6.46E-09 | 0.232268595 |
| MT-ND4 | 9.68E-09 | 0.229611345 |
| HSD17B2 | 1.07E-08 | 0.228922351 |
| MDK | 1.17E-08 | 0.228382918 |
| MEP1A | 1.70E-08 | 0.225874018 |
| CCL28 | 1.76E-08 | 0.225644115 |
| MT-ATP6 | 1.83E-08 | 0.225378178 |
| TXNIP | 2.83E-08 | 0.22240989 |
| CKB | 2.84E-08 | 0.222382459 |
| JCHAIN | 2.98E-08 | 0.222067131 |
| EPS8L3 | 3.29E-08 | 0.221385439 |
| MT-ATP8 | 3.31E-08 | 0.221343022 |
| MT1G | 3.38E-08 | 0.221203105 |
| GRN | 3.70E-08 | 0.220569663 |
| MUC13 | 4.69E-08 | 0.218935345 |
| GCC2 | 4.73E-08 | 0.218884742 |
| CD63 | 5.39E-08 | 0.217966376 |
| PPDPF | 5.74E-08 | 0.217535657 |
| SSFA2 | 7.09E-08 | 0.216050228 |
| USP53 | 8.03E-08 | 0.215174147 |
| TJP3 | 8.68E-08 | 0.214621377 |
| CAMK2N1 | 8.91E-08 | 0.214435924 |
| ANPEP | 1.26E-07 | 0.211952466 |
| GPA33 | 1.34E-07 | 0.211514423 |
| MVP | 1.59E-07 | 0.210274594 |
| AOC1 | 1.70E-07 | 0.20979218 |
| CLDN7 | 1.97E-07 | 0.208727287 |
| CDKN1A | 2.18E-07 | 0.207989378 |
| CCNL1 | 2.53E-07 | 0.206905402 |
| DDX5 | 2.64E-07 | 0.20657343 |
| CFDP1 | 3.56E-07 | 0.204377415 |
| MT1H | 3.59E-07 | 0.204319779 |
| RIOK3 | 3.59E-07 | 0.204305436 |
| CEACAM1 | 4.10E-07 | 0.203315014 |
| SRSF5 | 5.38E-07 | 0.201268443 |
| SPATS2L | 6.79E-07 | 0.199497996 |
| BTG1 | 6.82E-07 | 0.199464037 |
| HLA-B | 7.74E-07 | 0.198502067 |
| PTPRH | 8.53E-07 | 0.19775711 |
| ARL14 | 8.58E-07 | 0.197705149 |
| AMN | 1.19E-06 | 0.195198448 |
| TRIM31 | 1.29E-06 | 0.194521935 |
| CTSS | 1.44E-06 | 0.193691131 |
| HLA-G | 1.45E-06 | 0.193607561 |
| BCAS1 | 1.49E-06 | 0.193420468 |
| KRT8 | 1.53E-06 | 0.193215369 |
| TMEM54 | 1.74E-06 | 0.192175733 |
| SLC30A10 | 2.14E-06 | 0.19055214 |
| TCF7L2 | 2.65E-06 | 0.188816545 |
| RAP1B | 3.65E-06 | 0.186227972 |
| SCIN | 4.01E-06 | 0.185453966 |
| LIMA1 | 4.92E-06 | 0.183752178 |
| AQP8 | 5.52E-06 | 0.182808153 |
| SQSTM1 | 5.55E-06 | 0.182753805 |
| SLC17A4 | 5.62E-06 | 0.182648111 |
| FABP1 | 6.06E-06 | 0.18201916 |
| JOSD1 | 6.16E-06 | 0.181883756 |
| VAMP8 | 6.46E-06 | 0.181489278 |
| GLDN | 6.47E-06 | 0.181473761 |
| HIST1H1C | 6.74E-06 | 0.181134279 |
| LGALS3BP | 8.06E-06 | 0.179626954 |
| IGLC3 | 8.14E-06 | 0.179533608 |
| BSG | 9.16E-06 | 0.178536691 |
| CORO1B | 9.69E-06 | 0.178050696 |
| ENTPD8 | 1.06E-05 | 0.17729663 |
| CHMP2A | 1.09E-05 | 0.177027561 |
| SLC4A4 | 1.10E-05 | 0.176931666 |
| MISP | 1.21E-05 | 0.176126755 |
| LYPD8 | 1.37E-05 | 0.175056951 |
| PLAC8 | 1.55E-05 | 0.174000161 |
| VMP1 | 1.66E-05 | 0.173395229 |
| KLF4 | 2.02E-05 | 0.17167023 |
| CD164 | 2.05E-05 | 0.171525252 |
| MUC2 | 2.11E-05 | 0.171258917 |
| CLDN4 | 2.17E-05 | 0.17102352 |
| ISG20 | 2.40E-05 | 0.170120321 |
| MS4A12 | 2.46E-05 | 0.169888004 |
| MCL1 | 2.47E-05 | 0.169876072 |
| SECTM1 | 2.48E-05 | 0.169845705 |
| SEMA3B | 3.10E-05 | 0.167825294 |
| TM4SF20 | 3.45E-05 | 0.166854463 |
| LGALS9C | 3.82E-05 | 0.165931272 |
| FABP2 | 3.82E-05 | 0.165924236 |
| TCN2 | 4.14E-05 | 0.165183668 |
| CLTB | 4.32E-05 | 0.164789421 |
| LCN2 | 4.69E-05 | 0.164025554 |
| FBXW5 | 5.31E-05 | 0.162885093 |
| MGAM2 | 5.69E-05 | 0.162239235 |
| HLA-F | 6.35E-05 | 0.161208393 |
| TPT1 | 6.40E-05 | 0.161131522 |
| CIB1 | 7.07E-05 | 0.16019112 |
| DHRS9 | 7.44E-05 | 0.159704356 |
| HIST1H2AC | 7.71E-05 | 0.159370108 |
| SFN | 7.75E-05 | 0.159314598 |
| COL17A1 | 8.40E-05 | 0.158550049 |
| MT-CYB | 8.48E-05 | 0.158456616 |
| ACTN4 | 9.41E-05 | 0.15745406 |
| CDKN2B-AS1 | 0.000100748 | 0.15679888 |
| BTNL8 | 0.000106578 | 0.156254289 |
| MT1M | 0.000107094 | 0.156207366 |
| MT2A | 0.00011014 | 0.155935126 |
| PFKL | 0.000113402 | 0.155651249 |
| MYLK | 0.000118793 | 0.155198591 |
| MYO15B | 0.000141629 | 0.153473424 |
| NBL1 | 0.000176228 | 0.151303129 |
| LRP10 | 0.000181788 | 0.150992329 |
| CDKN2B | 0.000182665 | 0.15094411 |
| BHLHE41 | 0.000183755 | 0.150884455 |
| AKAP9 | 0.000184112 | 0.150865052 |
| SMPD1 | 0.000186747 | 0.150722508 |
| MUC4 | 0.000238193 | 0.148262533 |
| ELF3 | 0.000269647 | 0.146993827 |
| MAP1LC3B | 0.00027831 | 0.146668761 |
| MYO7B | 0.000291855 | 0.146178941 |
| GOLM1 | 0.000354349 | 0.144163119 |
| TRPM6 | 0.000358425 | 0.144043453 |
| HRASLS2 | 0.000362552 | 0.143923604 |
| KRTAP13-2 | 0.000370943 | 0.143683815 |
| CA2 | 0.000379529 | 0.143443617 |
| TSPAN3 | 0.000421208 | 0.142345113 |
| LAMB3 | 0.000425777 | 0.142230925 |
| TMC5 | 0.000433378 | 0.142043459 |
| LLGL2 | 0.000440446 | 0.141871879 |
| HHLA2 | 0.000465877 | 0.141274999 |
| CEMIP2 | 0.000483319 | 0.140882931 |
| SPINT1 | 0.000495123 | 0.140624977 |
| LSR | 0.000545616 | 0.139582478 |
| CDC42 | 0.000584456 | 0.138839899 |
| ITM2B | 0.000599834 | 0.138558491 |
| CES2 | 0.000722665 | 0.136524505 |
| DDX17 | 0.000804143 | 0.135345545 |
| PKN2 | 0.000840115 | 0.134859904 |
| PLA2G10 | 0.00099051 | 0.133017985 |
| ADTRP | 0.001060924 | 0.132243011 |
| TDP2 | 0.001092836 | 0.131907325 |
| PKIB | 0.001138079 | 0.131446574 |
| ABCG2 | 0.001169774 | 0.131133748 |
| CTSE | 0.001183722 | 0.13099856 |
| EPS8L2 | 0.001272083 | 0.130174726 |
| HSPA1A | 0.001322712 | 0.129726153 |
| TMPRSS2 | 0.001387141 | 0.129177615 |
| PDCD6IP | 0.001576889 | 0.127688452 |
| FER1L6 | 0.001580342 | 0.127662912 |
| APOBR | 0.001588666 | 0.127601549 |
| PLXNB2 | 0.001652203 | 0.127142682 |
| DSG2 | 0.001659366 | 0.127091969 |
| DNM2 | 0.001747239 | 0.126485689 |
| SLC15A1 | 0.001879349 | 0.125624859 |
| MYH14 | 0.001904855 | 0.125465076 |
| SLC40A1 | 0.001943161 | 0.125228752 |
| BEST4 | 0.002010708 | 0.124822238 |
| SCNN1B | 0.002199302 | 0.123750064 |
| CLDN23 | 0.002218732 | 0.123644436 |
| PSTPIP1 | 0.002427173 | 0.122561496 |
| EPS8 | 0.002475327 | 0.122323434 |
| CGN | 0.002625868 | 0.121605534 |
| SLC22A18 | 0.002786755 | 0.120878653 |
| SRRM2 | 0.002882216 | 0.120465245 |
| ISG15 | 0.003140527 | 0.119406087 |
| GGT1 | 0.003509625 | 0.118022566 |
| SPINK4 | 0.004182252 | 0.115810252 |
| RSRP1 | 0.004441668 | 0.115042461 |
| PDZD3 | 0.004447987 | 0.115024271 |
| CD68 | 0.00447305 | 0.114952348 |
| C2orf88 | 0.004483266 | 0.114923137 |
| EGLN3 | 0.004703486 | 0.114307618 |
| BMP3 | 0.005258397 | 0.112864946 |
| C1QTNF12 | 0.005283208 | 0.112803704 |
| EPCAM | 0.005286253 | 0.112796205 |
| ST14 | 0.005302754 | 0.112755639 |
| N4BP2L2 | 0.005371039 | 0.112588969 |
| BICDL2 | 0.005449358 | 0.11240014 |
| TNFSF10 | 0.006052815 | 0.111021977 |
| PTTG1IP | 0.006254391 | 0.110589094 |
| UBB | 0.006558304 | 0.109959554 |
| XDH | 0.006629652 | 0.109815561 |
| VAPA | 0.006657615 | 0.109759506 |
| CCDC68 | 0.006925433 | 0.109233071 |
| HEPH | 0.007120279 | 0.108861418 |
| OPTN | 0.00720502 | 0.108702616 |
| TCIRG1 | 0.007343663 | 0.108446374 |
| AKAP13 | 0.007457613 | 0.108238994 |
| ACAA2 | 0.007538338 | 0.108093788 |
| HDHD3 | 0.007921434 | 0.107423091 |
| P2RX4 | 0.008123327 | 0.107081213 |
| DDX3X | 0.008646592 | 0.106229224 |
| FOSB | 0.008652656 | 0.106219622 |
| MIR22HG | 0.009603365 | 0.104783787 |

**Table S3** Clinical characteristics of COVID-19 pneumonia patients

| ID | Gender | Age | Grade of COVID-19 |
| --- | --- | --- | --- |
| M001 | Male | 67 | Mild |
| M002 | Male | 81 | Mild |
| M003 | Male | 42 | Mild |
| M004 | Male | 80 | Mild |
| M005 | Female | 54 | Mild |
| M006 | Male | 43 | Mild |
| M007 | Female | 61 | Mild |
| M008 | Female | 47 | Mild |
| M009 | Female | 42 | Mild |
| M010 | Male | 27 | Mild |
| M011 | Female | 51 | Mild |
| M012 | Female | 27 | Mild |
| M013 | Female | 62 | Mild |
| M014 | Female | 52 | Mild |
| M015 | Female | 85 | Mild |
| M016 | Female | 36 | Mild |
| M017 | Female | 30 | Mild |
| M018 | Male | 26 | Mild |
| M019 | Female | 56 | Mild |
| M020 | Male | 51 | Mild |
| M021 | Male | 38 | Mild |
| M022 | Male | 62 | Mild |
| M023 | Female | 54 | Mild |
| M024 | Male | 53 | Mild |
| M025 | Female | 32 | Mild |
| M026 | Male | 53 | Mild |
| M027 | Male | 30 | Mild |
| M028 | Male | 35 | Mild |
| M029 | Female | 51 | Mild |
| M031 | Male | 35 | Mild |
| M032 | Male | 67 | Mild |
| M033 | Male | 60 | Mild |
| M034 | Female | 74 | Mild |
| M035 | Female | 67 | Mild |
| M036 | Male | 85 | Mild |
| M037 | Male | 63 | Mild |
| M038 | Male | 65 | Mild |
| M039 | Female | 51 | Mild |
| M040 | Female | 71 | Mild |
| M041 | Female | 72 | Mild |
| M042 | Male | 61 | Mild |
| M043 | Female | 51 | Mild |
| M044 | Female | 58 | Mild |
| M045 | Female | 56 | Mild |
| M046 | Female | 59 | Mild |
| M047 | Female | 68 | Mild |
| M048 | Male | 69 | Mild |
| M049 | Male | 51 | Mild |
| M050 | Female | 62 | Mild |
| M051 | Female | 61 | Mild |
| M052 | Female | 63 | Mild |
| M053 | Female | 51 | Mild |
| M054 | Male | 15 | Mild |
| M057 | Male | 61 | Mild |
| M058 | Male | 63 | Mild |
| M060 | Male | 73 | Mild |
| M061 | Male | 53 | Mild |
| M063 | Female | 78 | Mild |
| M064 | Female | 55 | Mild |
| M065 | Female | 70 | Mild |
| M067 | Male | 30 | Mild |
| M070 | Female | 57 | Mild |
| M071 | Male | 76 | Mild |
| M072 | Male | 54 | Mild |
| M073 | Female | 63 | Mild |
| M076 | Female | 69 | Mild |
| M077 | Female | 61 | Mild |
| M080 | Female | 60 | Mild |
| M081 | Female | 60 | Mild |
| M082 | Female | 65 | Mild |
| M083 | Male | 69 | Mild |
| M084 | Male | 70 | Mild |
| M085 | Male | 66 | Mild |
| M086 | Female | 70 | Mild |
| M087 | Female | 76 | Mild |
| M088 | Male | 39 | Mild |
| M089 | Male | 39 | Mild |
| M090 | Female | 55 | Mild |
| M091 | Male | 57 | Mild |
| M092 | Female | 69 | Mild |
| M093 | Male | 68 | Mild |
| M094 | Female | 50 | Mild |
| M095 | Male | 77 | Mild |
| M096 | Female | 65 | Mild |
| M097 | Male | 54 | Mild |
| M098 | Male | 48 | Mild |
| M099 | Female | 53 | Mild |
| M100 | Female | 44 | Mild |
| M101 | Male | 66 | Mild |
| M102 | Female | 51 | Mild |
| M030 | Female | 74 | Mild |
| M055 | Male | 60 | Mild |
| M056 | Male | 38 | Mild |
| M059 | Male | 86 | Mild |
| M062 | Male | 49 | Mild |
| M066 | Male | 86 | Mild |
| M068 | Male | 64 | Mild |
| M069 | Male | 49 | Mild |
| M074 | Male | 66 | Mild |
| M075 | Female | 74 | Mild |
| M078 | Female | 63 | Mild |
| M079 | Male | 62 | Mild |
| S002 | Male | 72 | Severe |
| S004 | Male | 69 | Severe |
| S005 | Male | 56 | Severe |
| S006 | Female | 58 | Severe |
| S010 | Female | 75 | Severe |
| S011 | Female | 61 | Severe |
| S012 | Female | 66 | Severe |
| S013 | Male | 73 | Severe |
| S016 | Male | 47 | Severe |
| S022 | Male | 83 | Severe |
| S028 | Male | 81 | Severe |
| S029 | Male | 56 | Severe |
| S031 | Male | 58 | Severe |
| S036 | Female | 80 | Severe |
| S043 | Female | 64 | Severe |
| S045 | Female | 80 | Severe |
| S046 | Male | 67 | Severe |
| S047 | Male | 61 | Severe |
| S001 | Female | 83 | Severe |
| S003 | Male | 81 | Severe |
| S007 | Female | 79 | Severe |
| S008 | Male | 66 | Severe |
| S009 | Female | 87 | Severe |
| S014 | Male | 57 | Severe |
| S015 | Male | 71 | Severe |
| S017 | Male | 62 | Severe |
| S018 | Male | 61 | Severe |
| S019 | Female | 69 | Severe |
| S020 | Female | 75 | Severe |
| S021 | Male | 62 | Severe |
| S023 | Male | 66 | Severe |
| S024 | Male | 85 | Severe |
| S025 | Male | 67 | Severe |
| S026 | Female | 80 | Severe |
| S027 | Male | 55 | Severe |
| S030 | Male | 73 | Severe |
| S032 | Male | 70 | Severe |
| S033 | Female | 62 | Severe |
| S034 | Male | 63 | Severe |
| S035 | Female | 58 | Severe |
| S037 | Male | 71 | Severe |
| S038 | Female | 93 | Severe |
| S039 | Female | 70 | Severe |
| S040 | Female | 63 | Severe |
| S041 | Male | 38 | Severe |
| S042 | Male | 77 | Severe |
| S044 | Male | 66 | Severe |
| S048 | Female | 86 | Severe |
| S049 | Female | 77 | Severe |
| S050 | Male | 51 | Severe |
