# Supplementary figures and images for: Single cell transcriptome revealed SARS-CoV-2 entry genes enriched in colon tissues and associated with coronavirus infection and cytokine production
Source: Signal Transduct Target Ther. 2020 Jul 8;5:121. doi: 10.1038/s41392-020-00237-0 (PMC7340775; doi:10.1038/s41392-020-00237-0)

Fig. S1

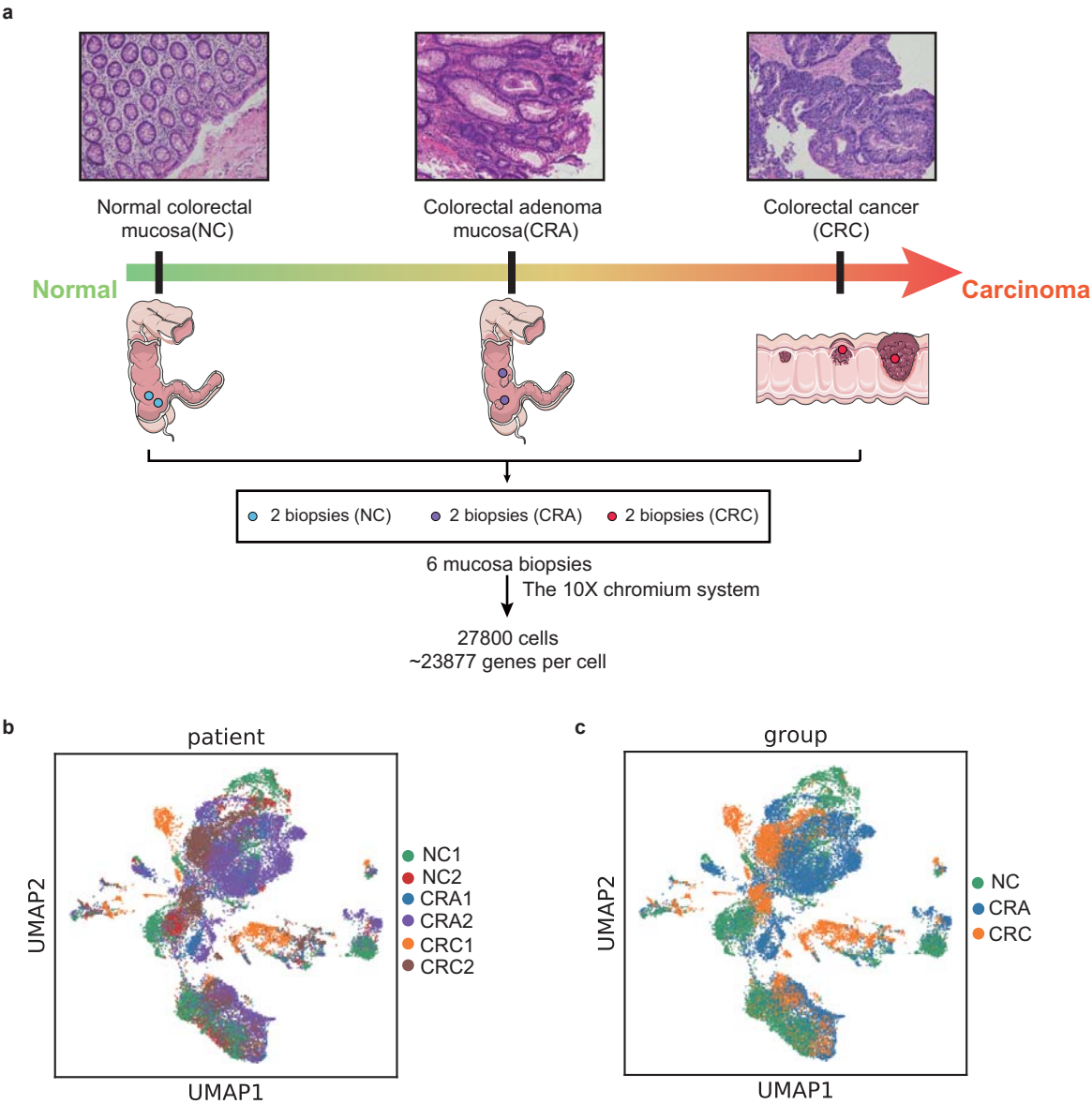

Fig. S2

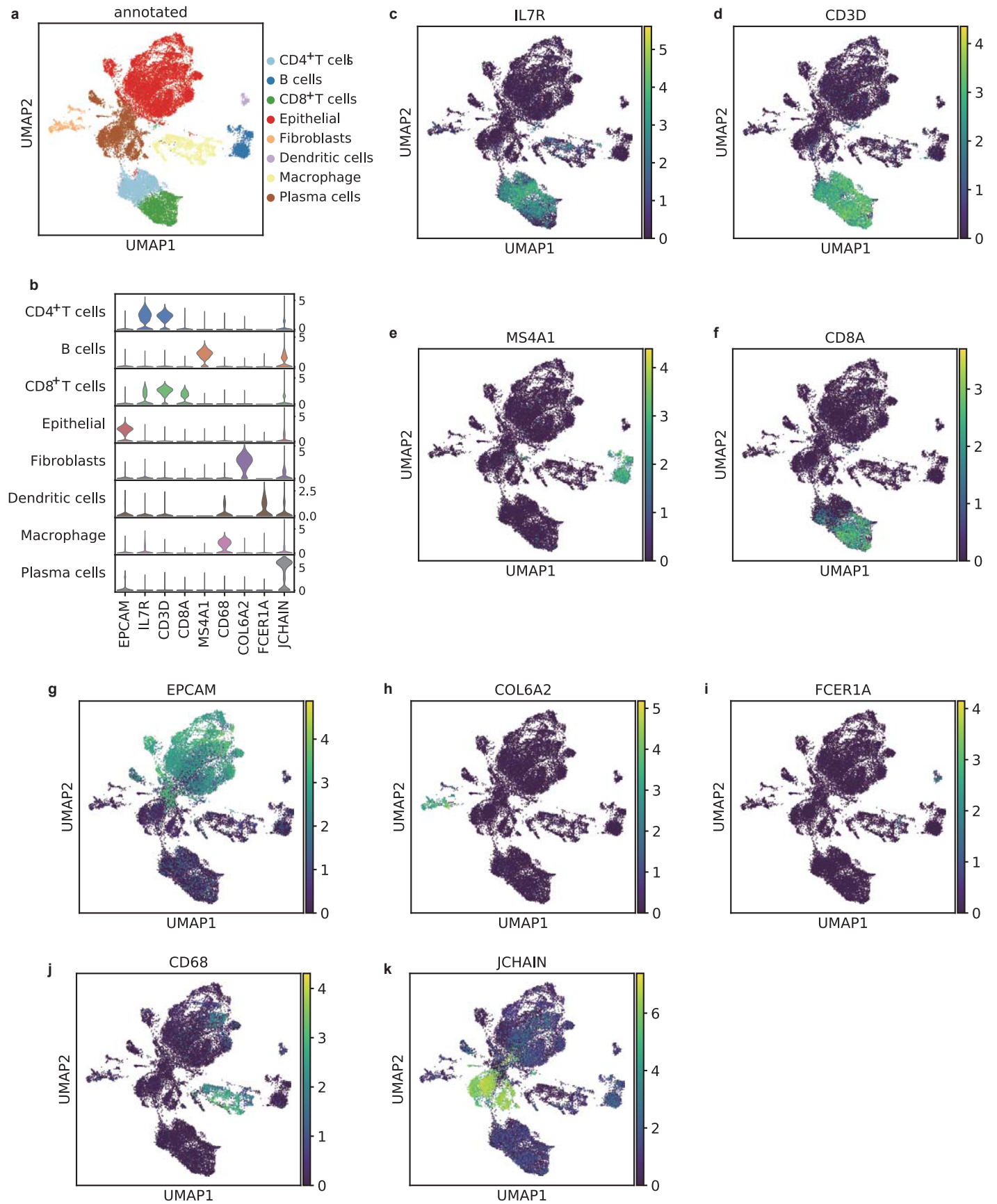

Fig. S3

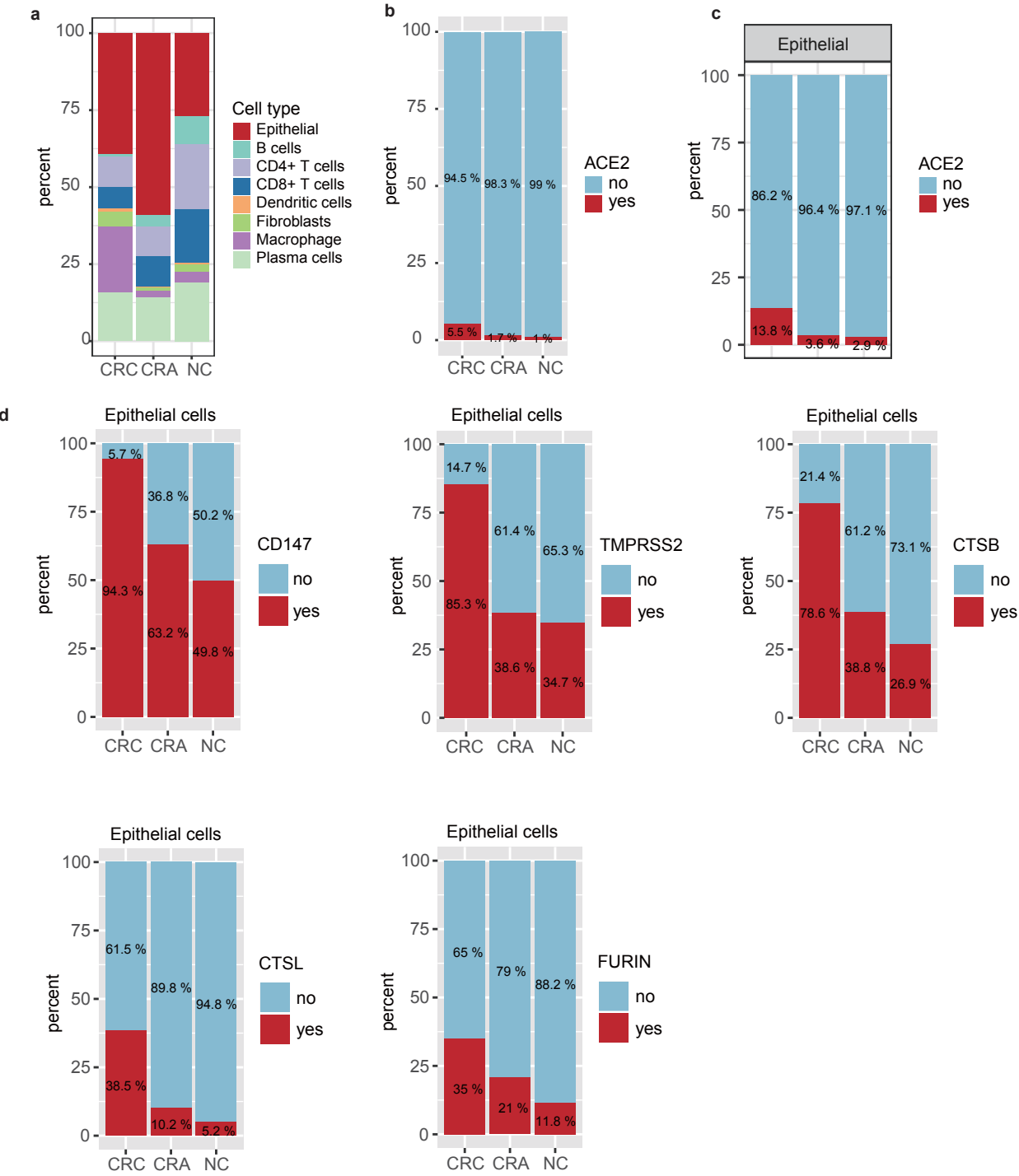

Fig. S4

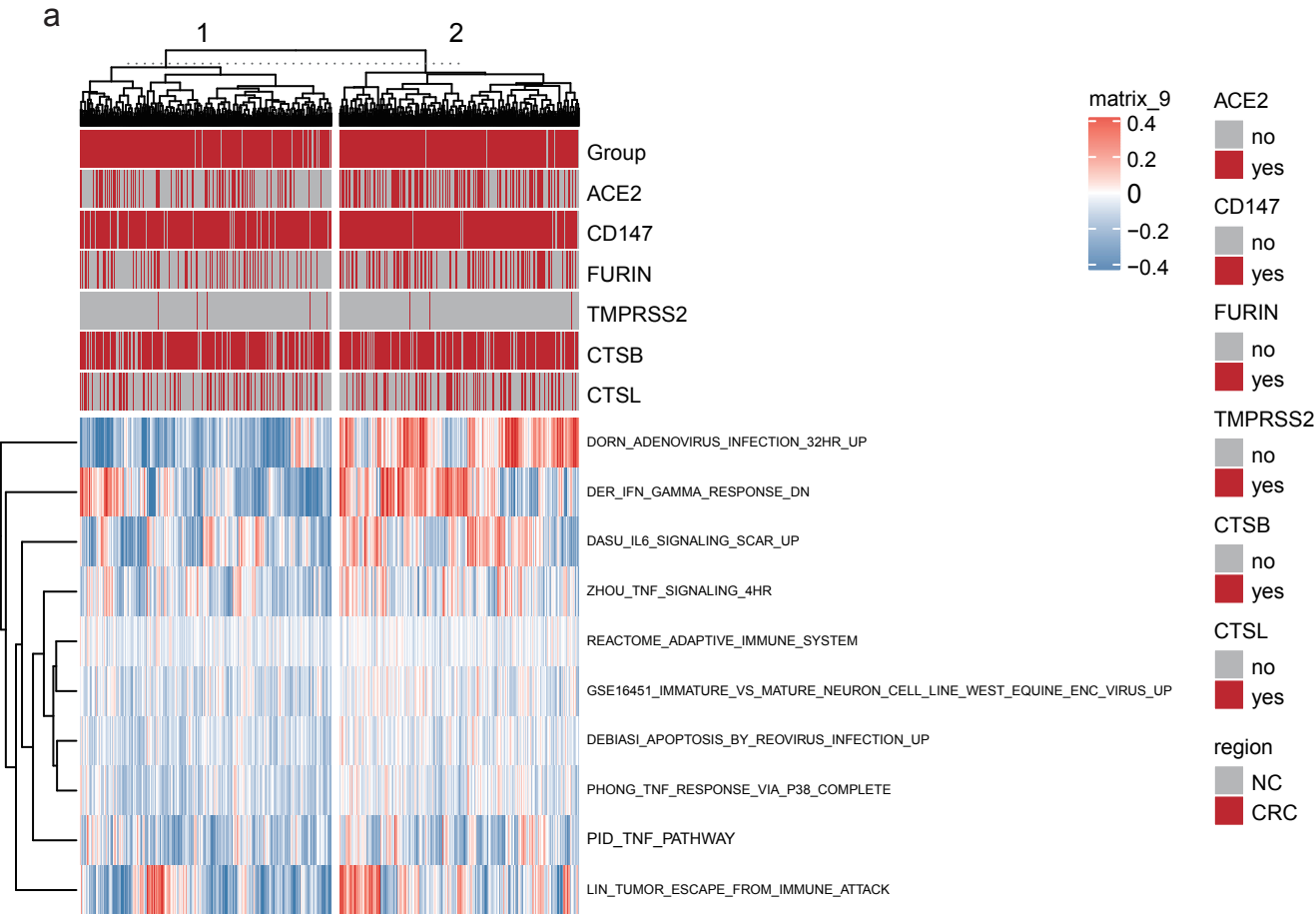

b

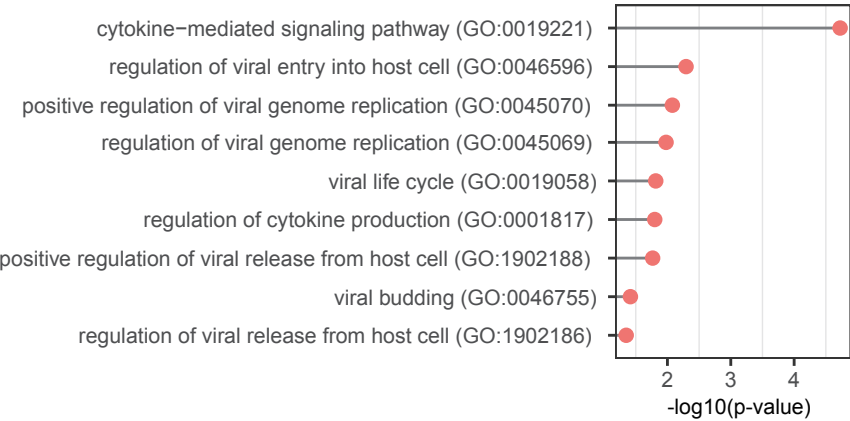

Supplement: Supplementary file 2 — Supplementary Figures [file 41392_2020_237_MOESM2_ESM.pdf]
